# From local control to immune modulation: hypofractionated radiotherapy as a backbone for cancer immunotherapy

**DOI:** 10.3389/fonc.2026.1750519

**Published:** 2026-02-05

**Authors:** Xiaoxuan Zhuang, Yao Sun, Dayong Zhuang

**Affiliations:** 1 Chongqing Medical University-University of Leicester Joint Institute, Chongqing Medical University, Chongqing, China; 2 Shandong University Scientific Journal Press, Shandong University, Jinan, China; 3 Department of Thyroid and Breast Surgery, The 960th Hospital of the People's Liberation Army of China Joint Logistics Support Force, Jinan, China

**Keywords:** cGAS–STING pathway, hypofractionated radiotherapy, immune checkpoint inhibitors, myeloid-derived suppressor cells, peri-radiotherapy window, tumour immune microenvironment

## Abstract

Hypofractionated radiotherapy (HFRT) is increasingly used not only for local tumour control but also for its immunomodulatory potential. By delivering higher doses per fraction over fewer sessions, HFRT improves local disease control and reshapes the tumour immune microenvironment. This review integrates preclinical, translational, and clinical evidence on the immunological effects of HFRT when combined with immune checkpoint inhibitors (ICIs) and other immune-based therapies. Available evidence indicates that HFRT induces immunogenic cell death and activates the cGAS-STING pathway, enhancing dendritic cell priming and CD8+ T-cell trafficking. These processes are most likely to translate into systemic antitumour activity when checkpoint blockade is delivered with an appropriate peri-radiotherapy window. Under these conditions, HFRT may facilitate immune conversion of selected “cold” tumours, particularly in combination with PD-1/PD-L1 blockade. Clinical outcomes remain heterogeneous across tumour types. Improved out-of-field responses and survival signals have been reported in non-small-cell lung cancer, head and neck squamous cell carcinoma, and triple-negative breast cancer, whereas tumours dominated by myeloid-driven or stromal suppression, such as pancreatic ductal adenocarcinoma, show limited benefit. From a clinical design perspective, effective HFRT-immunotherapy combinations require careful selection of fractionation, timing, and radiation geometry. Fractionation should preserve DNA sensing and dendritic-cell activation, checkpoint therapy should align with the peri-radiotherapy window, and radiation delivery should minimise immune suppression by sparing tumour-draining lymph nodes and limiting unnecessary low-dose exposure. HFRT can serve as a practical backbone for immune-based therapies when these variables are appropriately aligned.

## 1 Introduction

Radiotherapy (RT) remains a cornerstone of cancer care. Beyond its established role in achieving local tumour control through direct cytotoxicity effects, RT also reshapes the tumour microenvironment (TME) and can influence systemic antitumour immune responses (1, 2). Among RT strategies, hypofractionated radiotherapy (HFRT) delivers larger doses per fraction over fewer sessions. It is attractive not only for its practical efficiency but also for its distinct immune effects (3–5).

Preclinical and translational studies show that HFRT promotes immunogenic cell death and activates cyclic GMP–AMP synthase–stimulator of interferon genes (cGAS–STING) signalling. These changes support dendritic-cell cross-priming and increase CD8^+^ T-cell trafficking into the TME (6–8). Clinically, abscopal-like responses are reported more often when HFRT is combined with programmed cell death protein 1/ligand 1 (PD-1/PD-L1) blockade than when RT is used alone (2, 9–11).

Translation into routine practice has been uneven. Baseline tumour immunogenicity differs across disease sites, and clinical trials vary widely in fractionation schedules, sequencing of immune checkpoint inhibitors (ICIs), definition of systemic endpoints, and the extent of immune monitoring (12–15). Safety considerations further constrains trial design. In thoracic radiotherapy, pneumonitis risk must be anticipated (12, 16). In left-sided breast cancer, cardiac dose remains a central concern even with hypofractionated approaches (17). Accumulating evidence suggests that clinical benefit depends less on dose escalation alone than on how several modifiable treatment variables are aligned (18, 19). Fraction is a key determination. Very high single-fraction doses can induce TREX1 expression, accelerate cytosolic DNA clearance, and blunt cGAS–STING and type I interferon signalling. In contrast, moderate hypofractionation tends to preserve innate immune sensing and dendritic priming (20, 21). Timing represents a second determinant. Innate activation and dendritic-cell priming begin soon after the first fraction and evolve over subsequent days. Checkpoint blockade that overlaps this peri-RT window, broadly spanning from 48 hours to two weeks, is more likely to convert early priming into effective T-cells expansion and trafficking (8, 22). Geometry is a third determination. Radiation delivery incurs an immune cost through low-dose bath and blood-pool transit, and elective nodal irradiation can sterilise tumour-draining lymph nodes(TDLNs) that are required for efficient priming (23, 24). These spatial and temporal features are not captured by biologically effective dose (BED) alone and therefore need to be specified explicitly during trial design.

Two practical elements allow these principles to be tested across centres. The first is measurement. Early, prespecified sampling time points can capture both innate activation and early adaptive immune responses. A minimal immune panel should reflect interferon-linked signalling, dendritic-cell activation, early CD8^+^ T-cell dynamics, and simple indices such as absolute lymphocyte count (ALC) and monocyte-to-lymphocyte ratio (MonoLR) (12). Endpoints should capture responses in non-irradiated lesions to reflect systemic intent. For example, in microsatellite-stable colorectal cancer(MSCC), radiosurgery combined with tislelizumab produced partial or complete responses in 70% of non-irradiated lesionsin selected patients (25). The second element is planning and safety. Cardiopulmonary stewardship needs to be integrated into immune-aware planning. Geometry can be quantified with simple low-dose metrics that allow comparable across plans and reflect immune-relevant exposure (12, 24).

In this review, we focus on translating these biological insights into clinical actionable principles. We examine fractionation strategies that preserve DNA sensing, the placement of checkpoint blockade within the peri-RT window, and the role of radiation geometry as an immune modifier. We then consider endpoints, disease-specific contexts, safety constraints, and rational combination strategies when myeloid-driven immunosuppression limits response.

## 2 HFRT and the tumour immune microenvironment (TIME)

### 2.1 From local control to immune modulation: clinical anchors for HFRT

Clinical experience increasingly suggests that hypofractionated radiotherapy can contribute to systemic antitumour effects when it is combined with immune checkpoint inhibition, rather than acting solely as a local ablative modality. Across several tumour types, the addition of HFRT to immunotherapy-based regimens has been associated with improvements in objective response rate, disease control, progression-free survival, and overall survival (22, 26). Although the magnitude of benefit varies, these signals have been sufficiently consistent to support the concept of HFRT as an immune-modulating backbone in selected settings.

Importantly, these effects are not restricted to irradiated lesions. Even in palliative contexts, hypofractionated schedules delivered alongside PD-1 blockade have been associated with symptom relief and modest but reproducible activity in non-irradiated sites (27). While such responses are rarely dramatic, their recurrence across cohorts suggests engagement of systemic immune mechanisms rather than isolated local control.

Preoperative and window-of-opportunity studies provide more direct tissue-level evidence. In head and neck cancer, adding immune checkpoint blockade to short-course radiotherapy increases rates of major pathological response, indicating that immune activation can occur within the treated tumour under appropriate conditions (28). At the same time, clinical experience also defines clear limits. Stromally exclusionary tumours such as pancreatic ductal adenocarcinoma remain largely unresponsive to HFRT–immunotherapy combinations despite adequate local dosing, highlighting the dominant influence of tumour context and microenvironmental barriers (29, 30).

### 2.2 cGAS–STING kinetics and an immune-friendly fractionation

Fraction size directly influences how long cytosolic DNA persists after irradiation and how robust type I interferon signalling becomes. Very high single fractions induce exonucleases such as TREX1, leading to rapid degradation of cytosolic DNA and early termination of downstream immune sensing (31). Preclinical models demonstrated that single fractions above 12–15 Gy markedly increased TREX1 expression and reduced cytosolic DNA accumulation, whereas fractionated schedules such as 8 Gy × 3 preserved STING activation and interferon signalling (31–33). In contrast, moderate hypofractionated schedules are more likely to preserve cytosolic DNA, sustain cGAS–STING activation, and support interferon-driven immune engagement at the tumour site (20, 32).

This distinction is consistently observed in preclinical models. Regimens such as 8–10 Gy delivered in three fractions, or biologically comparable schedules, generate higher levels of cytosolic double-stranded DNA and stronger STING activation than single very high doses (34). From an immune perspective, fraction size therefore behaves as a biological variable rather than a purely physical one, a nuance that is not captured by conventional biologically effective dose calculations (33).

Clinical observations broadly align with these mechanistic data. In non–small-cell lung cancer(NSCLC), moderate hypofractionation combined with PD-1 blockade has been associated with higher response rates than immunotherapy alone (34). In breast cancer, hypofractionated regimens achieve durable local control with reduced acute toxicity compared with conventional schedules (35). By contrast, in tumours with pronounced stromal exclusion, such as pancreatic ductal adenocarcinoma, very high single fractions tend to underperform, underscoring the limits of immune engagement in hostile microenvironments (30).

### 2.3 The peri-radiotherapy window: definition, biology, and clinical implications

#### 2.3.1 Definition and temporal boundaries

The peri-RT window refers to a biologically defined period after hypofractionated radiotherapy, during which radiation-induced immune priming is most likely to evolve into effective adaptive antitumour immunity. Based on experimental kinetics and supportive translational observations, this interval generally spans from approximately 48 hours after treatment initiation to around 10–14 days thereafter (20–22, 36).

This window reflects the sequence of immune events initiated by radiation. Early danger signalling and type I interferon responses promote antigen release, dendritic-cell activation, and T-cell priming. Over time, counter-regulatory programmes, such as regulatory T-cell expansion, recruitment of myeloid-derived suppressor cells, and progressive T-cell dysfunction, become more prominent. Checkpoint blockade delivered within this interval is therefore more likely to act on an immune system that is actively primed, system rather than on a suppressed tumour microenvironment.

Thus, the peri-radiotherapy window should be viewed more as a biological opportunity, rather than a strict “concurrent” or “sequential” therapy window. While its precise boundaries may vary based on fractionation, tumour context, and host immune status, the underlying principle remains constant.

#### 2.3.2 Transition from innate immune activation to adaptive immune expansion

Hypofractionated radiotherapy initiates a rapid innate immune response, setting the stage for adaptive immunity. Within the first 24–72 hours, irradiated tumour cells undergo immunogenic stress and cell death, releasing damage-associated molecular patterns like calreticulin and high mobility group box 1(HMGB1) (20, 31, 32). At the same time, cytosolic DNA accumulation activates the cGAS–STING pathway and induces type I interferon production.

These signals drive dendritic-cell maturation and CCR7 upregulation, which allows antigen-loaded dendritic cells migrated to tumour-draining lymph nodes (21, 24). In lymphoid tissues, dendritic cells cross-present tumour antigens to naïve T cells, initiating clonal expansion and early differentiation of effector CD8^+^ T cells. This priming phase typically peaks within the first week after radiotherapy.

**Figure 1:**
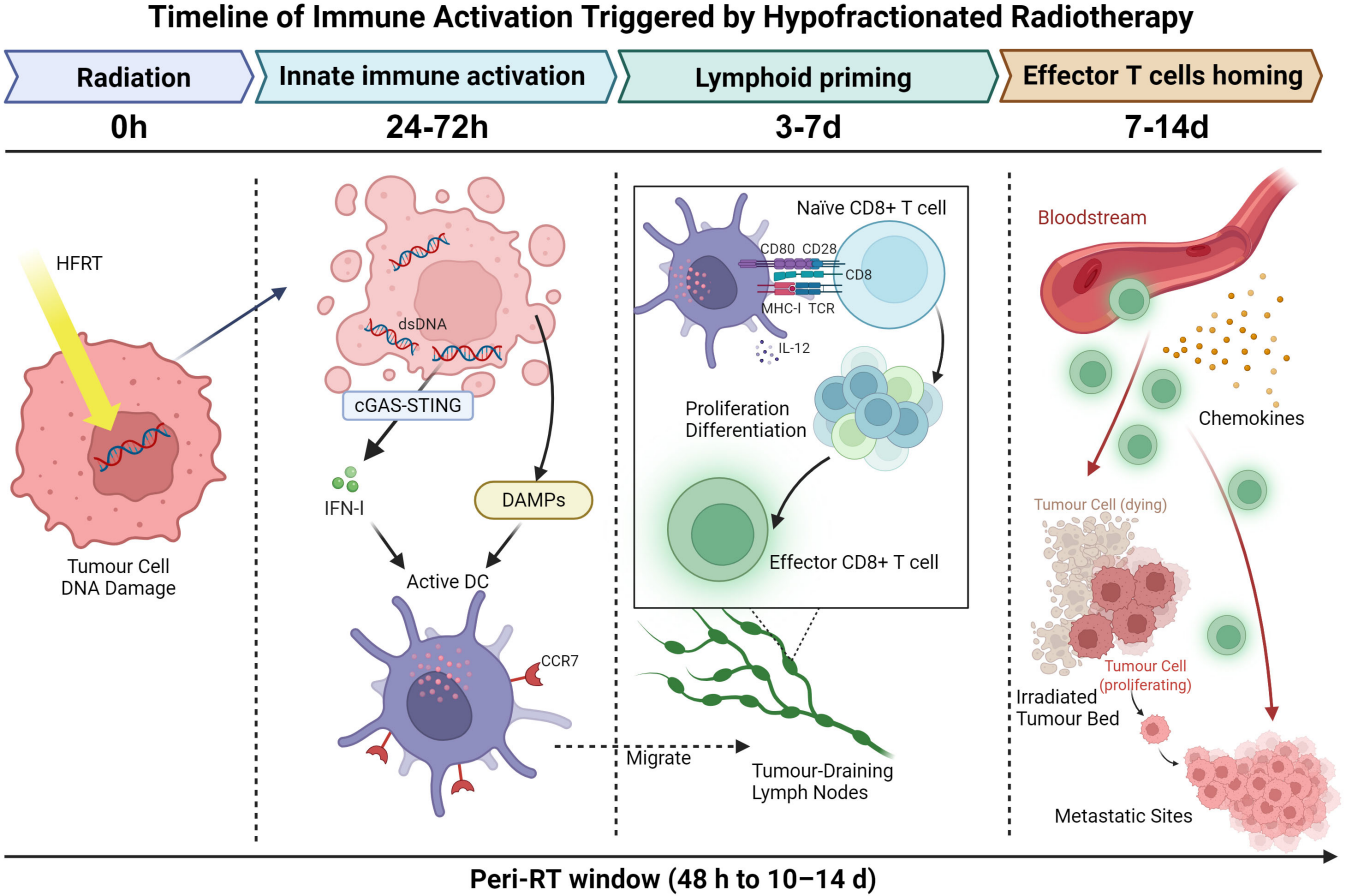
Temporal organisation of immune activation following hypofractionated radiotherapy. Hypofractionated radiotherapy induces tumour-cell DNA damage and cytosolic DNA sensing within the first 24–72 h, leading to type I interferon signalling, DAMP release, and activation of CCR7^+^ dendritic cells with migration to tumour-draining lymph nodes. Antigen cross-presentation and early CD8^+^ T-cell priming occur over days 3-7, followed by effector T-cell trafficking to the irradiated tumour bed and to non-irradiated metastatic sites over days 7-14, guided by chemokine and adhesion cues. The peri-radiotherapy window, spanning approximately 48 h to 10–14 d, is indicated along the timeline. DC: dendritic cell. Created in BioRender. Zhuang, X. (2026) https://BioRender.com/ertc4i1.

Between days 7 and 14, changes in adhesion molecules, chemokine gradients, and tumour vasculature facilitate trafficking of activated effector T cells back to the irradiated tumour bed and to non-irradiated metastatic sites (22, 36, 37). These immune events unfold in a defined temporal sequence, with early innate activation giving way to lymphoid priming and subsequent effector trafficking. For clarity, this timing is illustrated as a peri-radiotherapy window spanning approximately 48 h to 10–14 d, as shown in **Figure 1**. During this period, checkpoint inhibition can support T-cell function, prevent early exhaustion, and promote durable antitumour immunity rather than transient immune activation.

#### 2.3.3 Consequences of suboptimal sequencing

If checkpoint inhibition is delivered outside this window, synergy with radiotherapy may be weakened. Very early administration, before sufficient antigen release and dendritic-cell licensing, offers little for checkpoint blockade to act upon. Conversely, delayed treatment risks encountering an immune environment dominated by suppressive myeloid populations and exhausted effector cells.

These differences may help explain, at least in part, the variable outcomes reported across trials combining radiotherapy with immune checkpoint blockade. In these cases, lack of clinical benefit does not necessarily indicate failure of the combination itself, but rather mismatch with the immune kinetics at play.

#### 2.3.4 Clinical implications for scheduling and immune monitoring

Recognising the peri-radiotherapy window has important implications for trial design and clinical practice. Immune checkpoint inhibitors should ideally be initiated within a few days of the first hypofractionated dose and maintained throughout the first two weeks, coinciding with peak antigen presentation and T-cell expansion.

This strategy supports the use of two early immune monitoring time points, typically around 96 hours and 10–14 days after initiation (36, 37). These time points capture both innate immune activation and the emergence of adaptive responses. A practical monitoring panel may include peripheral lymphocyte counts, inflammatory ratios, interferon-related chemokines, markers of dendritic-cell activation, early CD8^+^ T-cell trafficking, and circulating myeloid-derived suppressor cells, as these are involved in myeloid-driven immunosuppression, which we discuss throughout in this review (38, 39).

### 2.4 Geometry, volume, and safety as determinants of immune benefit

**Figure 2:**
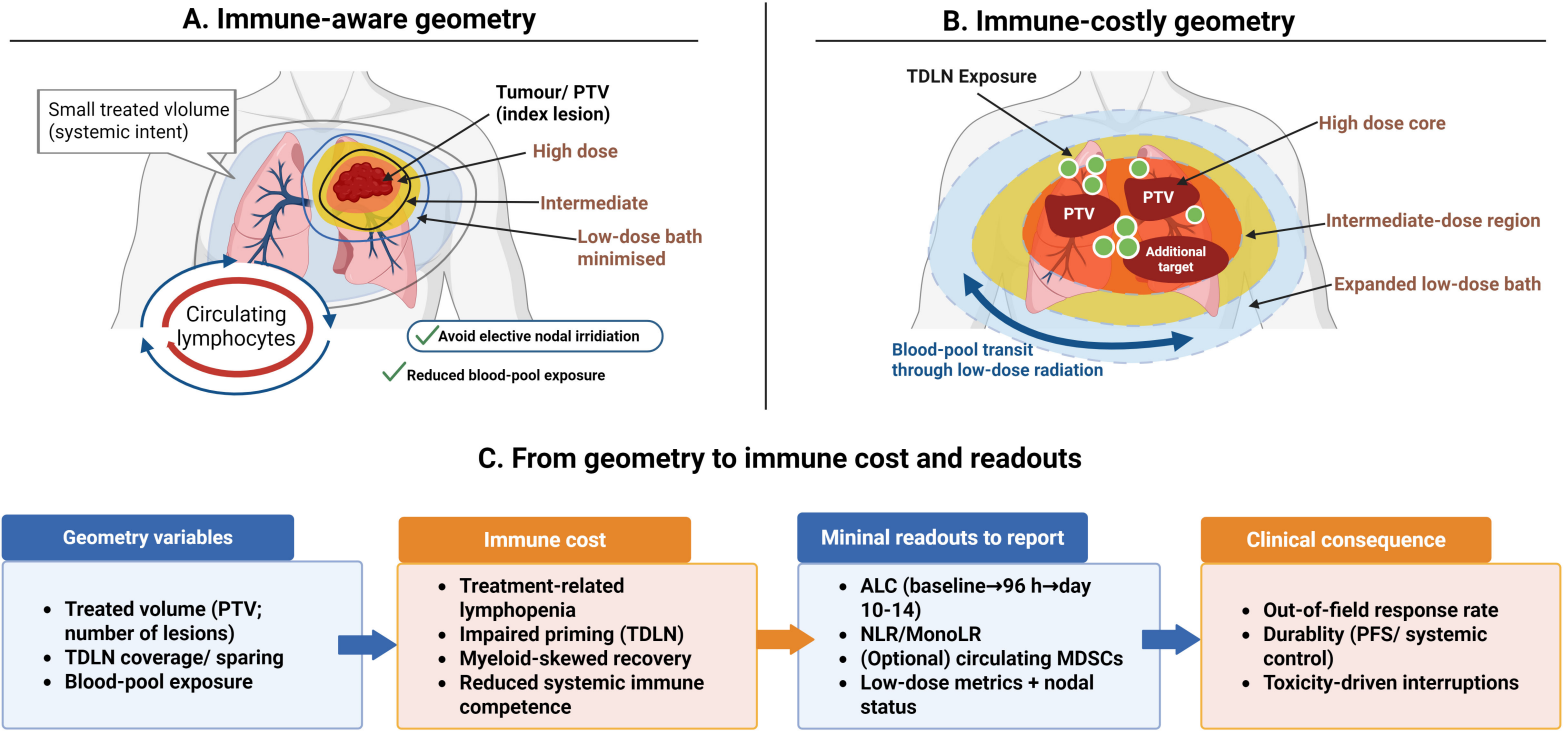
Geometry as an immune-cost lever in HFRT–immunotherapy programmes. **(A)** shows an immune-aware approach that prioritises a limited treated volume, minimises the low-dose bath, and spares tumour-draining lymph nodes when oncologically feasible, thereby reducing exposure of circulating lymphocytes. **(B)** illustrates a higher immune-cost configuration in which larger treated volumes, expanded low-dose exposure, and nodal coverage increase the risk of treatment-related lymphopenia and impaired priming. **(C)** outlines how geometry variables correlate with immune cost, minimal reporting readouts, and systemic endpoints assessed in non-irradiated lesions. Created in BioRender. Zhuang, X. (2026) https://BioRender.com/veo97j4.

In practice, field design often determines how much lymphocyte capacity is sacrificed to achieve local control. Circulating lymphocytes repeatedly pass through irradiated blood pools during beam delivery, and large low-dose volumes can reduce the viability of effector cells, even when target coverage is adequate (12, 40). Since systemic immune competence depends on preservation of circulating and regional effector pools, planning decisions directly influence whether HFRT can enhance immune-based therapies. Radiation geometry, therefore, provides a conceptual link between local dose delivery and systemic immune outcomes, as illustrated in **Figure 2**.

Immune-aware radiotherapy planning should use measurable geometric parameters rather than relying solely on dose prescription. Practical parameters include low-dose bath metrics, doses to tumour-draining lymph node regions, and exposure of haematopoietic organs such as spleen and bone marrow (24). These features capture aspects of immune preservation that are not reflected by BED metrics and should be specified explicitly in protocols aiming for systemic immune benefit.

Clinical experience further highlights the interaction between geometry and safety. In thoracic settings, radiotherapy delivered alongside PD-1 or PD-L1 blockade is associated with pneumonitis rates of approximately 30%-40% for all grades and around below 5% for grade 3 or higher events (12, 41). In breast cancer, whole-breast hypofractionation preserves oncological outcomes while reducing acute dermatitis, facilitating integration with systemic therapy. However, in left-sided disease, careful stewardship of mean heart dose remains essential given the dose-dependent increase in major coronary events (17, 41).

Across tumour sites, clinical observations support viewing radiation geometry as a biological determinant rather than a purely technical consideration. By avoiding unnecessary irradiation of irradiation of immune-relevant tissues and sparing tumour-draining lymph nodes when oncologically feasible, we help preserve immune competence and support systemic benefit (42, 43). These strategies can be incorporated into current planning workflows and should be considered integral to immune-informed radiotherapy.

## 3 The dual role of MDSCs in HFRT-induced TIME remodelling

### 3.1 Evidence that HFRT can act as an *in situ* vaccine across diseases

HFRT can induce effects beyond the treated lesion when its immune impact is appropriately integrated with systemic therapy. Reports of out-of-field responses suggest that such activity depends on alignment between radiation-induced antigen release and immune checkpoint blockade, typically within the peri-RT window. Under these conditions, HFRT may function as an *in situ* immune primer rather than a purely local treatment. The magnitude of systemic immune activity varies across tumour types, reflecting differences in tissue immune permissiveness. In head and neck squamous cell carcinoma(HNSCC), window studies have reported major pathological responses of 8%–17% with nivolumab alone and 20%–35% with the addition of ipilimumab (28). In the pre-operative setting for triple-negative breast cancer(TNBC), short-course RT within multimodality therapy achieves meaningful pathological complete response rates(pCR), and rates improve further with PD-1 blockade (29). In MSCC, a lesion-level analysis showed that radiosurgery plus tislelizumab produced partial or complete responses in most non-irradiated lesions, with a median progression-free survival(PFS) of 10.7 months (25). While other tumours remain resistant. In PDAC, the INFLUENCE trial tested a single high-dose SBRT of 15 Gy × 1 with nivolumab and ipilimumab. No objective responses were seen. The dense stroma likely blocked immune-cell infiltration and limited effective priming (30).

These observations support a practical rule. HFRT is most likely to act as an immune backbone when fractionation preserves DNA sensing, when treatment timing matches early immune activation, and when endpoints capture responses outside the irradiated field.

### 3.2 When inflammation invites suppression, choose partners by the failure mode

HFRT releases tumour antigens and activates type I interferon signalling. changes that would be expected to support antitumour immunity. At the same time, HFRT can recruit myeloid-derived suppressor cells(MDSCs) and skew macrophages toward suppressive phenotypes. This counter-regulation can limit distant tumour control and helps explain why “inflammation” does not always translate into durable immunity. This failure mode is well illustrated in oesophageal squamous-cell carcinoma(ESCC). Irradiation activates NF-κB, STAT3 and COX-2 pathways, upregulates PD-L1, and drives MDSC infiltration, a profile associated with variable benefit from PD-1 blockade alone (44).

Stromal barriers add further constraints. Tumours enriched for cancer-associated fibroblasts have been linked to NNMT activity with complement-driven MDSC expansion, and to SCG2 signalling through the LILRB4–STAT3 axis. These observations support pairing HFRT and checkpoint blockade with agents targeting COX-2, STAT3, CXCR2 or CCR2, depending on the dominant pathway (45, 46).

Low-dose radiotherapy can counter these suppressive loops. It improves perfusion, enhances antigen display, and reprograms myeloid cells. When paired with PD-1 blockade, LDRT provides reliable local control and symptom relief. Distant responses remain uncommon unless multiple sites are treated or the systemic backbone is strengthened (18, 47–50). When responses level off, as reported in some metastatic urothelial cancer cohorts, adding myeloid-targeted agents is more logical than repeating PD-1 therapy alone (51).

Partner choice should follow tumour biology. Prospective trials can stratify patients by inflammatory and myeloid features. COX-2 or STAT3 inhibition, or blockade CXCR2 or CCR2, may be appropriate when myeloid infiltration is prominent. Clinical interpretation should prioritise responses in non-irradiated lesions together with translational markers such as stromal and myeloid signatures (39, 45, 46). Simple indices, including baseline or early on-treatment MonoLR and NLR, can reflect myeloid skew, while radiation-induced PD-L1 upregulation may indicate checkpoint sensitivity. Where tissue is available, baseline cGAS–STING competence can help distinguish immune exclusion from impaired innate activation (38, 39).

### 3.3 Cold but convertible, proving systemic intent at the lesion level

These observations underscore the importance of testing systemic immune intent explicitly rather than inferring it from local control. Trial designs that focus on responses in non-irradiated lesions provide a more stringent assessment of immune-mediated benefit and reduce ambiguity between true systemic effects and cytoreductive outcomes at treated sites. In immunologically “cold” tumours, such lesion-level approaches offer a practical framework for determining whether HFRT has meaningfully altered the tumour-immune equilibrium (52).

### 3.4 Host metabolic and microbial modulators shaping systemic immune tone

In addition to tumour-intrinsic features and radiation-driven effects, host metabolic status and the gut microbiome can meaningfully influence the immune environment in which hypofractionated radiotherapy is delivered. Experimental and early clinical data suggest that microbial-derived metabolites affect myeloid cell behaviour, T-cell fitness, and sensitivity to immune checkpoint blockade, thereby shaping whether radiation-induced immune priming is maintained or curtailed (53, 54).

Among the pathways most consistently reported, alterations in tryptophan metabolism are particularly relevant. Activation of the indoleamine 2,3-dioxygenase (IDO1)–kynurenine–aryl hydrocarbon receptor axis promotes regulatory T-cell expansion and myeloid-mediated immunosuppression, which can blunt immune activation initiated by HFRT (55, 56). In contrast, short-chain fatty acids such as butyrate, produced by commensal bacteria including Roseburia species, enhance CD8^+^ T-cell cytotoxic function and improve responses to PD-1 blockade in colorectal cancer models, suggesting that microbial composition can influence adaptive immune competence after radiotherapy (54, 56).

Other metabolites appear to act in a tumour- and context-dependent manner. Trimethylamine N-oxide (TMAO), better known for its association with cardiovascular risk, has been shown to augment antitumour immunity in triple-negative breast cancer by enhancing effector T-cell function (57). Conversely, microbial-derived indole-3-lactic acid from Lactobacillus plantarum suppresses colorectal tumourigenesis through epigenetic regulation of CD8^+^ T-cell activity (58). Together, these findings underscore that host metabolic signals can either reinforce or counteract immune responses, depending on disease context and dominant immunological constraints (57).

At present, direct clinical modulation of the microbiome alongside HFRT remains exploratory. Nonetheless, variability in host metabolic and microbial profiles provides a plausible explanation for the heterogeneous immune responses observed among patients receiving otherwise similar radiotherapy–immunotherapy combinations. From a translational standpoint, incorporating host metabolic and microbial factors into mechanistically informed trial design may help refine patient stratification and improve interpretability of immune outcomes (53, 54, 57).

## 4 Immune checkpoint upregulation following HFRT: mechanisms and therapeutic windows

### 4.1 Eligibility and baseline stratification

When HFRT is combined with immunotherapy to pursue systemic benefit, clinical programmes should focus on tumours that can be immunologically modulated, rather than those suited only for local ablation. In practice, two baseline features are particularly informative. First, there should be evidence that checkpoint pathways are biologically relevant in the irradiated tumour. Inducible PD-L1 expression after radiation and preservation of the cGAS-STING/type I interferon axis are both associated with dendritic-cell recruitment, CD8^+^T-cell priming, and out-of-field responses when checkpoint blockade is delivered within the peri-radiotherapy window (20, 21). Where pretreatment tissue is available, documenting these features helps distinguish immune exclusion from impaired innate sensing when outcomes are negative. Second, host immune context should be considered at baseline. Peripheral inflammatory indices such as the neutrophil-to-lymphocyte ratio (NLR) and monocyte-to-lymphocyte ratio (MonoLR) are consistently associated with myeloid-skewed recovery and treatment-related lymphopenia (38, 59). In hepatocellular carcinoma, for example, smaller lymphocyte declines and a Th1-favoured baseline phenotype have been observed in patients with longer survival following locoregional therapy and immunotherapy (40). These readily available measures provide pragmatic stratification without reliance on specialised assays.

Eligibility should also be compatible with the intended timing of checkpoint therapy. Protocols designed to exploit peri-radiotherapy immune activation require patients who can start PD-1 or PD-L1 blockade within days of the first HFRT fraction and maintain treatment through the early post-radiotherapy interval. Safety considerations should be addressed at this stage, particularly in thoracic and left-sided breast settings, where pneumonitis risk and cardiac dose remain key constraints (12, 41).

### 4.2 Distinct immunobiology of PD-1/PD-L1 versus CTLA-4 blockade

PD-1/PD-L1 and CTLA-4 antibodies act at different stages of the antitumour immune response, and their interaction with HFRT is therefore not interchangeable. PD-1/PD-L1 blockade primarily restores effector T-cell function within peripheral tissues and the tumour bed, which fits well with HFRT-induced antigen release and interferon-responsive programmes (20, 21). In this setting, radiation-induced PD-L1 upregulation can represent an adaptive resistance mechanism that is directly targetable by PD-1 or PD-L1 inhibitors.

CTLA-4 blockade, by contrast, exerts its dominant effects during T-cell priming, influencing activation thresholds and regulatory T-cell dynamics within lymphoid compartments. As a result, combinations involving CTLA-4 antibodies may be more sensitive to factors such as antigen-presenting cell sufficiency, tumour-draining lymph node integrity, and the degree of baseline myeloid suppression (23, 28). These differences likely underlie the distinct toxicity profiles and variable efficacy observed across checkpoint classes when combined with radiotherapy.

In practice, selection of checkpoint strategy in HFRT-based regimens should reflect mechanistic alignment rather than class substitution. Programmes that fail to consider these differences risk mismatched combinations in which radiation-induced immune signals cannot be effectively amplified.

### 4.3 Timing and sequencing within the peri-RT window

Immune activation following HFRT evolves over a relatively short and biologically defined interval. Antigen presentation and early T-cell activation increase within days of treatment initiation and typically peak between approximately 48 hours and two weeks (22). Across HFRT-immunotherapy trials, heterogeneous outcomes are more often related to how effectively the peri-radiotherapy window is engaged than to whether radiotherapy and immunotherapy are simply combined.

Checkpoint blockade should be timed to overlap with radiation-induced immune priming, but not introduced so early that antigen release and dendritic-cell licensing are still incomplete. Conversely, delayed administration risks encountering an immune environment dominated by suppressive signals. Lesion-level assessment of non-irradiated sites provides a useful means of distinguishing true systemic immune effects from local cytoreduction, as demonstrated in MSCC and other settings (25, 52).

Interpretation of timing effects must also take radiation geometry into account. Differences in low-dose exposure and elective nodal irradiation can affect lymphocyte preservation and may confound the apparent efficacy of checkpoint blockade if not explicitly reported (23, 24, 42).

### 4.4 Endpoints and analyses beyond the irradiated field

Trials designed to modify systemic disease burden should measure outcomes outside the radiation field. In metastatic settings, overall response and progression-free survival should be reported alongside objective response and disease control rates in non-irradiated lesions. Lesion-level or multi-lesion designs reduce between-patient variability and strengthen claims of systemic benefit.

Published effect sizes can guide trial planning. In settings where out-of-field response rates are expected to increase from approximately 20% to 40% or where median progression-free survival may double, conventional two-arm designs are feasible (25–27). Stratification by inflammatory indices, irradiated volume, and the inducibility of PD-L1 after irradiation can further improve interpretability (38, 40).

### 4.5 Minimal reporting standards and safety context

Meaningful comparison across programmes requires consistent reporting of a small set of clinically relevant variables. A minimal dataset should include fractionation parameters, the timing of systemic therapy relative to radiotherapy initiation, the number and volume of irradiated lesions, and explicit documentation of responses in non-irradiated sites. Summary descriptors of low-dose exposure and nodal irradiation further contextualise immune preservation (23, 24, 42).

Where translational sampling is incorporated, paired early time points around 96 hours and 10–14 days after HFRT initiation can capture innate activation and emerging adaptive responses. Reporting of interferon-related signals, markers of immunogenic cell stress, dendritic-cell activation, early CD8^+^T-cell trafficking, PD-L1 dynamics, peripheral lymphocyte counts, and circulating MDSC subsets allows mechanistic interpretation without excessive logistical burden (21, 59–61).

Safety reporting should remain organ-specific and explicit. Pneumonitis risk in thoracic protocols and cardiac dose constraints in breast cancer remain central considerations when combining HFRT with immunotherapy (12, 41). Emerging delivery approaches such as proton therapy, FLASH irradiation, and spatially fractionated techniques should be described in sufficient detail to permit comparison across centres.

## 5 Clinical landscape: what generalises and what must be tailored

Across tumour types, clinical experience suggests that hypofractionated radiotherapy can support systemic immune effects when it is applied with clear immunological intent. The magnitude and consistency of benefit vary widely between diseases. What appears to generalise is not a specific regimen, but a set of shared design principles. By Contrast, what requires tailoring is dictated by tissue-specific barriers, dominant immunosuppressive programmes, and the treatment context. Key clinical patterns observed across tumour types are summarised in **Table 1**, illustrating how fractionation, partner selection, sequencing, and systemic readouts align in programmes reporting immune-mediated activity.

**Table 1:** Reproducible design patterns for using hypofractionated radiotherapy as an immune primer across tumour types.

Disease/setting	HFRT backbone (typical)	Checkpoint strategy	Primary systemic readouts	Key design notes
Non–small-cell lung cancer (oligo-/metastatic)	8–10 Gy × 3 (or biologically comparable)	PD-1/PD-L1	Responses in non-irradiated lesions; PFS/OS signals	Align with peri-RT window; minimise lung/heart low-dose exposure
Head and neck squamous cell carcinoma (window or neoadjuvant)	Short-course HFRT	PD-1 ± CTLA-4	Major pathological response	Exploit early priming; tailor nodal coverage to preserve TDLNs
Triple-negative breast cancer	Hypofractionated whole-breast or partial-breast RT	PD-1/PD-L1	Pathological response; relapse-free survival	Cardiac dose stewardship; immune effects depend on baseline TILs
Microsatellite-stable colorectal cancer (metastatic)	Lesion-directed SBRT	PD-1 with immune amplification	Responses in non-irradiated lesions (heterogeneous)	Often requires multi-site or myeloid-aware strategies
Pancreatic ductal adenocarcinoma	SBRT or short-course HFRT	PD-1–based combinations	Limited systemic activity	Dominant stromal and myeloid suppression constrain benefit
Melanoma (oligometastatic/metastatic)	SBRT to selected lesions	PD-1/PD-L1 ± CTLA-4	Abscopal-like responses; survival signals	Immune-responsive context; geometry and timing remain critical

HFRT, hypofractionated radiotherapy; SBRT, stereotactic body radiotherapy; OS, overall survival;PD-1, programmed cell death protein 1; PD-L1, programmed death-ligand 1. Patterns summarise reproducible clinical design features rather than prescriptive regimens. “± CTLA-4” indicates that dual checkpoint blockade has been explored in selected settings, typically where enhanced priming is desired. Relative sequencing refers to alignment with radiation-induced immune activation (peri-radiotherapy window).

Head and neck window-of-opportunity studies show that short-course protocols can induce measurable immune changes at the tissue level. Major pathological response rates range from approximately 8% to 17% with nivolumab alone and increase to about 20%–35% with the addition of ipilimumab (28). These findings are particularly relevant for older or frail patients who are often underrepresented in pivotal trials. In locally advanced cutaneous squamous-cell carcinoma, a low-frequency HFRT schedule (700–800 cGy weekly for 6–8 fractions) combined with cemiplimab achieved an objective response rate of 57.1%, including 19% complete responses, with acceptable toxicity and fewer treatment interruptions than standard hypofractionation (62). Together, these data define a realistic range for the degree of immune modulation achievable in head and neck settings.

In non–small-cell lung cancer, a more consistent and robust systemic response has been reported. Notably, gut-targeted antibiotics combined with stereotactic body radiotherapy have shown promise in enhancing outcomes for medically inoperable early-stage NSCLC (63), while in the PEMBRORT phase II trial, 8 Gy × 3 HFRT combined with pembrolizumab was associated with a median overall survival of 15.9 months, compared with 7.6 months for pembrolizumab alone, and abscopal responses were observed in a subset of patients (34). Preclinical and translational data support the biological plausibility of these findings, showing increased neoantigen release and expansion of antigen-specific CD8^+^ T cells following moderate hypofractionation (34).

Early triple-negative breast cancer shows a comparable pattern. Short-course radiotherapy integrated into multimodality care is associated with meaningful pathological complete response, with further improvement when combined with anti–PD-1 therapy (12). Whole-breast hypofractionation maintains tumour control while reducing acute skin toxicity, facilitating combination with systemic treatment. In left-sided disease, careful management of mean heart dose remains essential because of the dose-dependent risk of major coronary events (17, 41).

By contrast, translation remains limited in stromally exclusionary or myeloid-dominant tumours. Pancreatic ductal adenocarcinoma is a clear example. In the INFLUENCE study, stereotactic body radiotherapy at 15 Gy × 1 combined with nivolumab, ipilimumab, and an influenza vaccine produced no objective responses (30). Microenvironmental constraints are likely central, particularly cancer-associated fibroblast programmes that promote myeloid-derived suppressor cell recruitment and restrict T-cell access (53), while preclinical study indicate that dual blockade of LIF and PD-L1 can enhance the antitumour efficacy of SBRT in murine PDAC models (64). In this context, combining moderate HFRT (6 Gy × 5) with NNMT-targeted strategies reduced MDSCs, improved CD8^+^ T-cell access, and increased disease control compared with HFRT alone (53, 65), supporting the need for stroma- and myeloid-directed partners rather than escalation of radiotherapy or checkpoint blockade alone. Oesophageal squamous-cell carcinoma shows a related biology, with radiation-induced activation of NF-κB and STAT3 pathways and recruitment of suppressive myeloid populations, providing a rationale for combinations that target dominant inhibitory signals (44).

Hepatocellular carcinoma highlights the importance of field design and host context. In treatment sequences incorporating transarterial chemoembolisation, stereotactic body radiotherapy, and immunotherapy, larger irradiated volumes are associated with deeper lymphocyte depletion and poorer outcomes. By contrast, smaller lymphocyte declines and a Th1-favoured baseline correlate with longer survival (40). These observations support immune-sparing approaches and underscore the value of routinely reporting immune-relevant geometric parameters, including lesion number, target volume, and low-dose exposure patterns (23, 24, 42).

Across programmes reporting systemic immune effects, several common themes emerge, including fractionation that sustains immune engagement, geometry-conscious planning to limit unnecessary immune cost, and partner selection guided by dominant biological barriers. Explicit assessment of non-irradiated lesions strengthens claims of systemic intent and improves comparability across tumour types (57, 59, 66).

## 6 Operationalising immune modulation

### 6.1 Baseline selection and stratification

When hypofractionated radiotherapy is combined with immunotherapy with the aim of achieving systemic benefit, patient selection must go beyond suitability for local control. In practice, baseline biological features often determine whether HFRT can meaningfully prime an immune response or remains a purely cytoreductive intervention.

At the tumour level, two features are particularly informative. First, PD-L1 should be inducible after irradiation rather than constitutively absent, as adaptive upregulation reflects preserved interferon responsiveness. Second, the cGAS–STING/IFN-I axis should remain functionally intact, since failure of innate sensing limits downstream immune priming regardless of fractionation or timing (20, 21). Where tissue is available, baseline assessment can help distinguish immune exclusion due to physical or stromal barriers from impaired innate activation, allowing negative outcomes to be interpreted more accurately.

Host immune context also contributes to stratification. Simple peripheral inflammatory indices, including the NLR and MonoLR are consistently associated with myeloid-skewed recovery and treatment-related lymphopenia (38, 59). Clinical experience in hepatocellular carcinoma illustrates that smaller post-radiotherapy lymphocyte declines together with a Th1-favoured baseline phenotype associate with longer survival (40, 41).

### 6.2 Integrated decisions on fractionation, geometry, and safety

After patient selection, whether HFRT contributes to systemic immune control is largely determined by how the treatment plan is constructed. Fractionation, radiation geometry, and safety should be considered as a single integrated framework, as together they define the immune cost of local control.

Fraction size influences whether radiation-induced tumour injury sustains immune activation or prematurely suppresses it. Hypofractionated schedules that preserve cytosolic DNA sensing and avoid excessive exonuclease induction are more likely to maintain antigen presentation and downstream immune engagement (20, 21). Fractionation should not be selected solely on oncological convenience, but also with regard to immune preservation.

Radiation geometry is equally critical because it shapes lymphocyte exposure. Extensive low-dose irradiation, elective nodal coverage, and large target volumes increase the risk of depleting circulating and regional immune cell pools. By contrast, limiting the low-dose bath and sparing tumour-draining lymph nodes, when oncologically appropriate, helps preserve immune competence (23, 24). In practice, this may involve prioritising an index lesion when systemic intent is primary and documenting immune-relevant dosimetric features such as low-dose volume exposure and marrow or splenic dose (23, 24, 42).

Safety considerations define the ceiling for immune-oriented radiotherapy. Treatment plans that balance tumour control with preservation of cardiopulmonary, haematopoietic, and lymphoid function are more likely to allow uninterrupted systemic therapy and to sustain any immune-mediated benefit initiated by HFRT (12, 41). Optimising fractionation, geometry, and safety in isolation is insufficient, while immune preservation depends on their alignment.

### 6.3 Emerging radiation technologies in immune-aware planning

Most principles guiding immune-oriented HFRT derive from experience with photon-based radiotherapy. Newer delivery platforms, however, offer additional opportunities to reduce immunological cost while maintaining tumour control.

Proton therapy allows tighter confinement of dose deposition, reducing irradiation of surrounding normal tissues, circulating blood pools, and lymphoid structures. Dosimetric comparisons in thoracic and abdominal malignancies consistently show lower low-dose exposure with proton-based techniques, a feature that is directly relevant when preservation of lymphocyte reserves is a treatment objective (23, 24, 40, 42). Given the association between treatment-related lymphopenia and inferior outcomes in patients receiving immune checkpoint inhibitors, this lymphocyte-sparing profile provides a clinical rationale for considering proton therapy in immune-oriented strategies (12, 40).

Clinical evidence combining proton therapy with immunotherapy remains limited. Early-phase experiences and retrospective series suggest that proton-based hypofractionated regimens can be delivered safely alongside immune checkpoint inhibition without unexpected toxicity, while better preserving peripheral lymphocyte counts compared with photon-based approaches (67, 68). Whether these dosimetric advantages translate into superior systemic immune efficacy remains to be determined.

Ultra-high dose-rate irradiation (FLASH radiotherapy) and spatially fractionated techniques, such as lattice or GRID radiotherapy, have also attracted interest. Preclinical studies suggest that these approaches may spare normal tissues and circulating immune cells while maintaining tumour control, but their immunological effects in combination with immunotherapy are not yet defined in routine clinical settings (69–71).

Emerging technologies should be viewed as extensions of immune-aware planning rather than substitutes for it. Their potential value lies in further reducing unnecessary immune injury. In clinical practice, their role should be evaluated using the same criteria applied to conventional HFRT, with attention to alignment of fractionation, geometry, and safety with immune objectives, supported by immune monitoring and assessment of systemic endpoints (67, 68).

### 6.4 Mechanism-guided combination strategies

In clinical practice, the benefit of combining HFRT with immunotherapy largely depends on whether the chosen systemic partner addresses the dominant immunosuppressive constraint of a given tumour type. Adding agents without a clear mechanistic rationale may increase toxicity while offering little additional immune control.

Tumours characterised by prominent myeloid-driven immunosuppression, such as ESCC and hepatocellular carcinoma (HCC), illustrate this point. In these settings, HFRT can activate inflammatory signalling pathways but also promotes recruitment of myeloid-derived suppressor cells, which limits effective immune priming. Interventions that restrict myeloid trafficking or function, including CXCR2 or CCR2 blockade, have been shown to reduce suppressive cell influx, improve CD8^+^ T-cell access, and enhance the efficacy of radiotherapy-based combinations (34, 40, 44).

By contrast, immunologically “cold” tumours such as microsatellite-stable CRC often fail at the level of immune amplification rather than suppression. In these cases, additional immune stimulation may be required to convert radiation-induced antigen release into effective adaptive responses. Cytokine-based strategies, including IL-2 or GM-CSF, provide one approach by supporting T-cell expansion and dendritic-cell recruitment, and have shown encouraging activity in selected settings when integrated with HFRT (25, 34).

Across tumour types, mechanism-guided design favours removal of dominant inhibitory influences over indiscriminate escalation of checkpoint inhibition. Attention should also be paid to treatments that undermine immune activation, including avoiding using prolonged high-dose steroids and unnecessary broad-spectrum antibiotics during peri-radiotherapy period (72, 73).

Viewed in this way, hypofractionated radiotherapy is less of an isolated cytotoxic intervention and more of a flexible platform, with its systemic impact depending on biological context. Identifying the main immune limitation in each tumour type and selecting partners that specifically target this constraint offers a more reliable path to durable systemic benefit than relying on trial-and-error combination strategies.

## 7 Discussion and conclusion

Current evidence supports a pragmatic view of HFRT as a potential immune-modulating backbone rather than a purely local intervention. Across tumour types, systemic signals are most consistently reported when radiation delivery, immune engagement, and clinical evaluation are conceptually aligned. These effects do not emerge from any single variable but from the convergence of fractionation choices, immune context, and tumour-specific barriers.

Clinical experience across disease settings suggests that systemic immune signals are most reliably observed when radiotherapy is deliberately integrated into immune-based strategies, while disease-specific constraints continue to shape both efficacy and safety. At the same time, these signals do not generalise uniformly. Pancreatic ductal adenocarcinoma exemplifies a resistant phenotype, where dense stroma and myeloid-mediated barriers limit the ability of HFRT and checkpoint blockade alone to generate systemic control (30, 53). In this context, improved outcomes appear to depend on partners that directly address stromal and myeloid barriers, rather than escalation of radiation dose or checkpoint therapy in isolation. Comparable logic applies to oesophageal squamous-cell carcinoma, where inflammatory and myeloid programmes shape response and motivate combinations that target dominant inhibitory pathways (44–46).

Radiation geometry and safety act as boundary conditions for immune modulation. Strategies that limit unnecessary lymphocyte exposure, spare tumour-draining lymph nodes when oncologically acceptable, and constrain low-dose irradiation of immune-relevant tissues help preserve systemic immune competence (23, 42). Clinical observations in hepatocellular carcinoma underscore this principle, linking larger irradiated volumes to deeper lymphocyte depletion and poorer outcomes (40).

These findings support viewing HFRT as a tunable platform rather than a fixed intervention. Fractionation sets the tone of immune sensing. Geometry then determines how much immune capacity is lost in achieving local control, while partner selection ultimately decides whether these signals translate into durable systemic benefit. There is therefore no single regimen that applies across settings. Instead, HFRT-based immunotherapy requires context-specific tailoring guided by the dominant biological constraints of each tumour type.

Future progress will likely depend on more integrative biomarker stratification beyond broad inflammatory ratios, the development of regimens adapted to vulnerable populations, and trial designs that explicitly test systemic intent through assessment of non-irradiated lesions. When approached in this way, HFRT is not simply an adjunct to immunotherapy but a configurable backbone that can expose antigen, engage immune sensing, and support systemic control when treatment design respects both tumour biology and immune preservation. Aligning these principles across diseases may help translate promising signals into more consistent clinical benefit.

### 7.1 Limitations

This review is narrative rather than systematic, and heterogeneity in fractionation schedules, systemic partners, and clinical endpoints limits direct comparison across studies. Many available datasets are retrospective or single-arm, introducing potential selection and immortal-time biases. Translational correlates linking HFRT-induced immune changes to patient-level outcomes remain underpowered, and head-to-head comparisons of biologically similar fractionation schemes are scarce. These limitations should be considered when interpreting conclusions and highlight the need for prospective, mechanism-informed studies.
